# Retinal vessel metric analysis of type 1 diabetes mellitus in OCT angiography

**DOI:** 10.3389/fmed.2025.1562809

**Published:** 2025-06-13

**Authors:** Nianfeng Tang, Na Su, Zetian Zhang, Keren Xie, Qiang Chen, Wen Fan, Junjie Shan, Songtao Yuan

**Affiliations:** ^1^Department of Ophthalmology, The First Affiliated Hospital of Nanjing Medical University, Nanjing, China; ^2^School of Computer Science and Engineering, Nanjing University of Science and Technology, Nanjing, China; ^3^Department of Ophthalmology, Changzhou Third People’s Hospital, Changzhou, China; ^4^Department of Ophthalmology, The Affiliated Taizhou People’s Hospital of Nanjing Medical University, Taizhou, China

**Keywords:** diabetic retinopathy, type 1 diabetes mellitus, OCTA, vessel density, deep learning, vessel metric changes of T1DM

## Abstract

**Purpose:**

To investigate retinal vascular characteristics in type 1 diabetes mellitus (T1DM) patients at different stages via optical coherence tomography angiography (OCTA).

**Design:**

A retrospective observational study.

**Method:**

63 T1DM patients (110 eyes) who underwent OCTA (3*3 mm) examination and 40 age-matched healthy individuals (79 eyes) were included. A deep learning model was used to segment the retinal arteries and veins on OCTA images, and the vascular metrics in the macular area, including fractal dimension (FD), vessel diameter index (VDI), vascular length fraction (VLF), vascular tortuosity, and vessel density (VD) in different zones (fovea, superior parafoveal, inferior parafoveal, nasal parafoveal, and temporal parafoveal) were calculated.

**Results:**

In accordance to the diagnostic criteria for diabetic retinopathy (DR), T1DM patients were classified into groups. There were 12 individuals (19 eyes) in the NPDR group, all of whom exhibited non-proliferative DR, and 51 (91 eyes) in the NDR (non-DR) group. The NDR group was divided into 2 subgroups according to the duration of T1DM, with 28 people (49 eyes) having a duration of less than 5 years and 23 people (42 eyes) having a duration of 5 years or more. Built-in metrics of OCTA showed that the VD in each zone of NPDR group was significantly lower than that of control group and NDR group (all *p* < 0.05). In addition, the VD of the inferior parafoveal vein (*P* = 0.022) and superior parafoveal artery (*P* = 0.03) were significantly decreased in the NPDR group. Comparing the VD among NDR subgroups and control group, the VD of the superficial inferior parafovea of T1DM patients of early stage (less than 5 years) was significantly lower than that of normal people (*p* < 0.05), while the VD of the inferior parafoveal artery increased (*P* = 0.012).

**Conclusion:**

We employed a deep learning vessel segmentation model to analyze the changes in arterial and venous metrics in OCTA images of T1DM patients. Early damage of NPDR to large vessels occurs in the inferior parafoveal vein and the superior parafoveal artery. In patients without DR, the arterial VD of the inferior parafovea has a compensatory increase.

## 1 Introduction

Type 1 diabetes mellitus (T1DM), an autoimmune disorder characterized by pancreatic β-cell destruction and absolute insulin deficiency, affects approximately 8.75 million individuals worldwide (1, 2). Diabetic retinopathy (DR), one of the most common microvascular complications of diabetes, is a leading cause of visual impairment and blindness in adults (3). Patients with T1DM have a higher risk of developing DR and tend to develop DR at an earlier age than those with type 2 diabetes mellitus (3, 4). Consequently, early screening and prediction of DR in T1DM patients are of utmost importance.

Optical coherence tomography angiography (OCTA) is a noninvasive retinal vascular imaging technique that can clearly depict the microvascular structure and blood flow of the retina and choroid. This enables better tracing of arterioles and venules, making OCTA ideal for investigating vascular changes in the preclinical and initial stages of DR (5–7). With the rapid development of artificial intelligence (AI) image processing technology in recent years, vessel segmentation by deep learning models can extract vascular structural features in images, reduce noise interference in OCTA images, and provide abundant vascular quantification indices, such as vessel diameter, length, and tortuosity (8–10). This method conducts a more in-depth analysis of OCTA images and identifies subtle changes imperceptible to the retinal (11, 12).

Traditional views hold that the pathogenesis of DR is initiated by microvascular lesions, and as DR progresses, lesions gradually involve large vessels (13, 14). However, recent findings have shown that larger retinal vessels also exhibit changes, such as increased diameter and blood flow velocity in the early stages of DR (15, 16). Moreover, there are differences in the effects of blood glucose fluctuations on retinal arteries and veins. In the early stages of diabetes, it mainly affects the permeability of the arterial side of retinal capillaries and precapillary arterioles (17, 18), whereas in the later stage of DR, it begins to affect venules (19). Currently, most analyses of retinal vascular parameters in DR primarily focus on microvascular structure and blood flow, with relatively insufficient attention paid to large vessels. This may lead to an incomplete understanding of the pathogenesis of DR and potentially affect the accuracy of OCTA in predicting the onset of DR.

Therefore, this study aimed to utilize a deep learning vessel segmentation model to assist in observing the retinal vascular characteristics of T1DM patients at various stages on OCTA images and to explore the early retinal vascular changes in T1DM patients.

## 2 Materials and methods

This was a retrospective, observational study. The study was ethical (2019-SR-304) and in accordance with the tenets of the Declaration of Helsinki.

### 2.1 Participants

This study enrolled 110 eyes of 63 T1DM patients and 79 eyes of 40 healthy individuals who underwent OCT angiography (3 mm × 3 mm) at Jiangsu Province Hospital from July 2018 to August 2019.

Inclusion criteria were (1) diagnosis of type 1 diabetes, (2) older than 18 years of age, and (3) OCTA images with a quality of Q8 or higher. Exclusion criteria were (1) diagnosis of proliferative diabetic retinopathy (PDR) or diabetic macular edema (DME); (2) eyes with a history of intraocular surgery; (3) myopia > −6D, hyperopia > +1D, or axial length of the eye greater than 26 mm; and (4) eyes with ocular disease other than DR (e.g., age-related macular degeneration, glaucoma, retinal vein occlusion).

The clinical characteristics and OCTA images were obtained from the recruited patients. The clinical characteristics included age, gender, and duration of diabetes mellitus (defined as the time between the date of diagnosis and OCTA imaging). T1DM patients were divided into two groups based on the diagnostic criteria for diabetic retinopathy (DR): 12 (19 eyes) in the NPDR group, all of whom had non-proliferative diabetic retinopathy (NPDR) on fundus examination (20), and 51 (91 eyes) in the NDR (non-DR) group. The NDR group was divided into 2 subgroups according to the duration of T1DM, with 28 people (49 eyes) having a duration of less than 5 years and 23 people (42 eyes) having a duration of 5 years or more.

### 2.2 OCT angiography imaging

The OCTA device used was Angiovue OCTA (version 2018.0.0.14; rtvue xr avanti; Optovue, Fremont, CA, USA). The device uses an 840 nm light source with a scan rate of 70,000 scans per second. The scanning mode was selected as a preset macular area of 3 mm × 3 mm to quantitatively evaluate the vascular metrics of the retina and choroid in the macular area. En face images of the superficial capillary plexus (SCP) was defined as the layer from the internal limiting membrane (ILM) to the inner plexiform layer (IPL), and the deep capillary plexus (DCP) was defined as the layer from the outer border of the IPL to the outer plexiform layer (OPL), and the fovea region was defined as a circular area with a radius of 0.5 mm centered on the central fovea (21). The parafoveal region was defined as a ring between the fovea and the outer circle with a radius of 1.5 mm. The parafoveal regions were automatically divided into 4 equal quadrants (temporal, nasal, inferior, and superior).

### 2.3 Vascular segmentation and metrics

**FIGURE 1:**
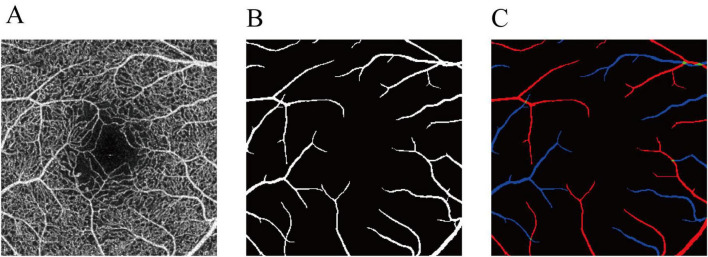
Annotation of the arteries and veins in OCTA-500. **(A)** OCTA inner-retinal projection. **(B)** The large vessel label. **(C)** The final artery-vein label. Red represents arteries. Blue represents veins. Green represents the arteriovenous junctions.

We employed IPN-V2 (22) for vessel segmentation. IPN-V2 is a 3D-to-2D image segmentation method that utilizes a fast projection module during the 3D projection stage to achieve 3D feature compression. The method then completes the pixel-level segmentation task during the 2D segmentation stage. We utilized 3 mm OCTA volume from OCTA-500 dataset (22) for training. The input data consisted of the entire OCTA volumetric dataset (with dimensions 304 × 640 × 604), and the output data represented the segmented arteriovenous vessels in the projection image (Figure 1).

Functional indices derived from OCTA images include mean direction angle change (MDAC), length of the curve (LC), Total Squared Curvature normalized by the blood vessels’ chord length (TSC-LX), Total Squared Curvature normalized by the blood vessels’ arc length (TSC-LC), vessel density (VD), fractal dimension (FD), vessel diameter index (VDI), and vessel length fraction (VLF). The definitions are as follows:


(1) MDAC=

$$\frac{1}{\left(t_{l\,e\,n\,g\,t\,h}-2*s\,t\,e\,p\right)}\,\sum_{n=s\,t\,e\,p}^{t_{l\,e\,n\,g\,t\,h}-s\,t\,e\,p}a\,r\,c\,c\,o\,s\,U\,V\,\left(P_{n-s\,t\,e\,p},P_{n}\right)\cdot U\,V\,\left(P_{n},P_{n+s\,t\,e\,p}\right)$$

Where, *t*_*length*_ stands for the length of the particular track and *UV* means unary vector. MDAC (Equation 1) measures tortuosity by averaging the change of angles calculated at reasonable discrete steps along the blood vessel (23).


(2) $$\begin{array}{c} L_{C}=\sum_{i=1}^{n-1}\sqrt{{\left(x_{i}-x_{i+1}\right)}^{2}+{\left(y_{i}-y_{i+1}\right)}^{2}} \end{array}$$

(3) $$\begin{array}{c} \mathrm{TSC}\,\left(S\right)=\int_{t_{0}}^{t_{n}}C\,{\left(t\right)}^{2} \end{array}$$

(4) $$\begin{array}{c} \mathrm{TSC}\,\mathrm{normalized}\,\mathrm{by}\,\mathrm{LX}=\frac{\mathrm{TSC}\,\left(S\right)}{\mathrm{LX}\,\left(S\right)} \end{array}$$

(5) $$\begin{array}{c} \mathrm{TSC}\,\mathrm{normalized}\,\mathrm{by}\,\mathrm{LC}=\frac{\mathrm{TSC}\,\left(S\right)}{\mathrm{LC}\,\left(S\right)} \end{array}$$

TSC-LX and TSC-LC (Equations 2–7) are two different tortuosity indicators which are estimated by normalizing the total squared curvature by vessel arc length and vessel chord length, respectively (23). The length of the chord and the curvature at point t are expressed as:


(6) $$\begin{array}{c} L_{X}=\sqrt{{\left(x_{n}-x_{1}\right)}^{2}+{\left(y_{n}-y_{1}\right)}^{2}} \end{array}$$

(7) $$\begin{array}{c} C\,\left(t\right)=\frac{x^{\prime }\,\left(t\right)\,y^{\prime \prime }\,\left(t\right)-y^{\prime }\,\left(t\right)\,x^{\prime \prime }\,\left(t\right)}{{\left[x^{\prime }\,{\left(t\right)}^{2}+y^{\prime }\,{\left(t\right)}^{2}\right]}^{\frac{3}{2}}} \end{array}$$

(8) $$\begin{array}{c} \mathrm{VD}=\frac{\sum V}{N} \end{array}$$

(9) $$\begin{array}{c} \mathrm{VDI}=\frac{\sum V}{\sum S} \end{array}$$

(10) $$\begin{array}{c} \mathrm{VDI}=\frac{\sum S}{N} \end{array}$$

where, V represents the pixels identified as the vessel area, and N denotes the total number of pixels in the entire area. S signifies the pixels identified as the vessel skeleton. VD (Equation 8) is calculated as the proportion of the vessel-occupied area to the overall area (24, 25). VDI and VLF (Equations 9, 10) reflect the average caliber and length of the blood vessels (26, 27). FD is computed using a box-counting technique on the vessel skeleton image (28), which captures the geometric complexity of the retinal blood vessels.

### 2.4 Statistical analysis

Statistical analyses were performed using SPSS 27.0 (SPSS, Inc., Chicago, IL, USA). The measurement data were expressed as mean ± standard deviation, and the count data were expressed as frequency (percentage). Data were tested for normality using the Kolmogorov–Smirnov test, and the one-way ANOVA test was used to compare between groups of measures that met the normal distribution. The LSD method was used when the variance was uniform and Tamhane’s T2 method was used when the variance was not uniform. Non-parametric tests were used to compare between groups of measured information that did not conform to a normal distribution, and the chi-square test was used to compare between groups of count information. All *P*-values were two-tailed and considered significant if *p* < 0.05.

## 3 Results

Baseline characteristics of the enrolled patients showed no statistically differences in age, gender among the three groups in the control, NDR, and NPDR groups (all *p* > 0.05) (Table 1).

**TABLE 1:** Baseline characteristics of participants.

		Control (*n* = 70)	NDR (*n* = 91)	NPDR (*n* = 19)	*P*-value
Age, year		25.00 ± 8.249	23.63 ± 9.556	29.92 ± 12.362	0.12
Gender	Male, *n* (%)	16 (40.0)	26 (51.0)	7 (58.3)	0.424
Eye	OS, *n*, (%)	37 (52.9)	46 (50.5)	10 (52.6)	0.955
	OD, *n*, (%)	33 (47.1)	45 (49.5)	9 (47.4)	
Duration of T1DM, year		–	5.479 ± 5.427	11.458 ± 6.747	0.002**

T1DM, type 1 diabetes mellitus; NPDR, non-proliferative diabetic retinopathy; NDR, non-diabetic retinopathy. **p < 0.01.

**FIGURE 2:**
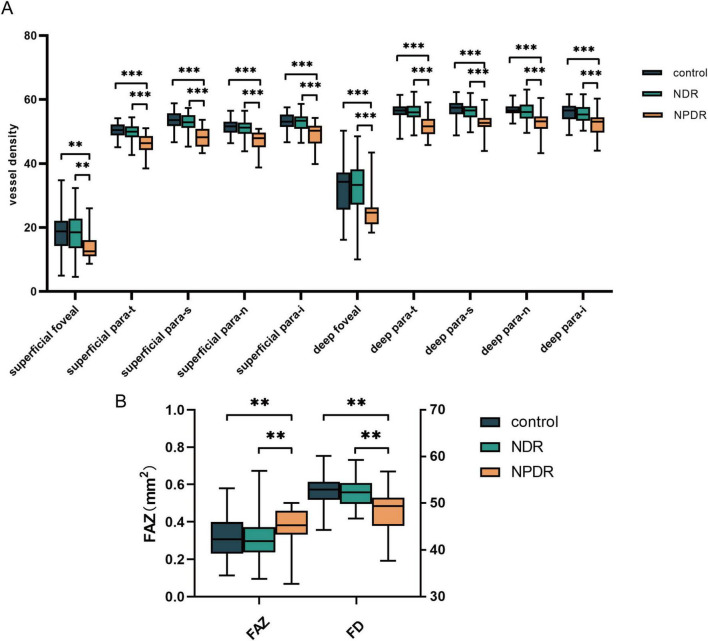
Analysis of OCTA equipment built-in metrics. **(A)** The significant differences of VD among control group, NDR group and NPDR group. **(B)** The differences of FAZ and FD among groups mentioned above. NDR, non-diabetic retinopathy; NPDR, non-proliferative diabetic retinopathy; FAZ, foveal avascular zones; FD, fractal dimension. **p < 0.01; ***p < 0.001.

We first analyzed the built-in metrics of the OCTA equipment in the three groups (Supplementary Table 1). A significant decrease in the VD of all zones (foveal, para-t, para-s, para-n, and para-i) in both the deep and superficial capillary plexus, an enlargement of the FAZ, and a decrease in the FD of the vessels were observed in the NPDR group compared with the control and NDR groups (all *p* < 0.05) (Figure 2).

**FIGURE 3:**
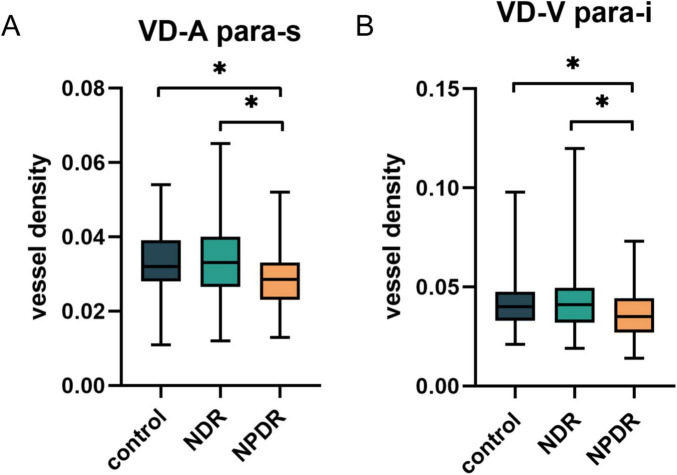
The differences in VD of superior parafoveal arteries **(A)** and inferior parafoveal veins **(B)** among control group, NDR group and NPDR group after vessel segmentation. NDR, non-diabetic retinopathy; NPDR, non-proliferative diabetic retinopathy. *p < 0.05.

To further investigate the alterations in the large vessels among the three groups, we segmented the arteries and veins in the OCTA images using deep learning-assisted vessel segmentation and calculated their VD separately, including the metrics of MDAC, LC, TSC-LX, TSC-LC, VDI, and VLF (Supplementary Table 2). The findings revealed that modifications in the vascular metrics of the large vessels had already appeared in the NPDR group, as evidenced by a decrease in the VD of the inferior parafoveal arteries (*p* = 0.03) and the superior parafoveal veins (*p* = 0.02), whereas no significant changes in the VDI, VLF, or tortuosity (including MDAC, LC, TSC-LX, TSC-LC, and FD) of the vessels were detected (all *p* > 0.05) (Figure 3).

In addition, we explored the presence of altered vessel metrics in patients with NDR of different disease durations. We further divided the NDR group into 2 subgroups according to T1DM duration, < 5 years group included 28 patients (49 eyes) with a disease duration of < 5 years, and ≥ 5 years group enrolled 23 patients (42 eyes) with a disease duration of 5 years or more. No significant differences were observed in age and gender among the control, < 5 years, and ≥ 5 years groups (all *p* > 0.05) (Table 2).

**TABLE 2:** Baseline characteristics of subgroups.

		Control (*n* = 70)	< 5 years (*n* = 49)	≥ 5 years (*n* = 42)	*P*-value
Age, year		25.00 ± 8.249	21.36 ± 9.056	26.39 ± 9.609	0.105
Gender	Male, *n* (%)	16 (40.0)	15 (53.6)	11 (47.8)	0.534
Eye	OS, *n*, (%)	37 (52.9)	25 (51.0)	21 (50.0)	0.954
	OD, *n*, (%)	33 (47.1)	24 (49.0)	21 (50.0)	
Duration of T1DM, year		–	1.658 ± 1.220	10.130 ± 4.890	–

T1DM, type 1 diabetes mellitus.

**FIGURE 4:**
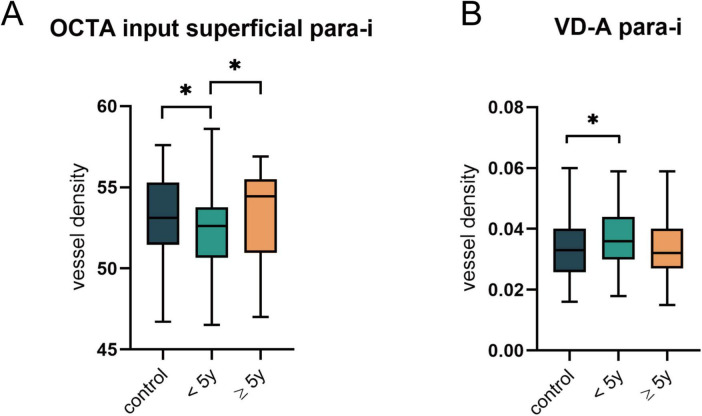
Comparison of OCTA built-in parameters and VD after vascular segmentation in the NDR subgroups. **(A)** The significant differences in the VD of superior parafoveal vascular in superficial plexus as indicated by OCTA built-in parameters. **(B)** Changes in arterial VD of superior parafovea after vessel segmentation. *p < 0.05.

Analysis of the built-in metrics of the OCTA equipment (Figure 4A) revealed that the VD of the inferior parafovea in the superficial capillary plexus was significantly lower in the group of T1DM patients with a disease duration of less than 5 years than in both the control group and T1DM patients with a disease duration of more than 5 years (all *p* < 0.05). However, no significant alterations were detected in the FAZ, FD, or zones (foveal, para-t, para-s, para-n, and para-i) in the DCP (Supplementary Table 3).

Additionally, we analyzed the vascular metrics of the images after segmentation of arteries and veins (Supplementary Table 4) and discovered that the VD of arteries in the inferior parafoveal zone was significantly elevated in T1DM patients with a disease duration of less than 5 years (*p* = 0.035) (Figure 4B). At this stage, the arteries and veins of the other zones did not exhibit variations. Similarly, no significant differences were found in VDI, VLF, and vessel tortuosity-related parameters (all *p* > 0.05).

## 4 Discussion

DR is initiated by microvascular lesions, featuring the loss of pericytes of capillaries as the earliest pathological manifestation of DR (29). Decreased VD in various regions of the SCP and DCP has been documented in the early stages of DR, even prior to the development of fundus lesions (7), with more pronounced alterations in VD on the arterial side of the capillaries and enlargement of the FAZ (30). Gardiner et al. (29) found that at the microaneurysm formation stage, precapillary arterioles already exhibited localized diameter enlargement and loss of vascular smooth muscle cells, as evidenced by staining and electron microscopy. This implies that large vascular changes occur during the early stages of DR. Venous beading and looping gradually emerged with the progression of DR, suggesting worsening of the lesion. Ishibazawa et al. (31) indicated that capillary nonperfusion could progress from the arterial side toward the venous side, with smaller areas of nonperfusion tending to be arterial-adjacent, whereas larger areas of nonperfusion are more likely to be venous-adjacent. This distinction is crucial since DR affects blood vessels in specific patterns. By separately analyzing arteries and veins, models can identify nuanced changes specific to DR (32).

Literature is scarce on separately analyzing retinal arterial and venous vascular metrics in T1DM patients. Palochak et al. (15) analyzed metrics such as arterial and venous blood flow in the adaptive optics scanning laser ophthalmoscope (AOSLO) images of patients with NPDR and found a compensatory increase in arterial blood flow in the early stage of DR. Cheung et al. (17) prospectively analyzed fundus photographs of patients with T1DM and revealed that arterial dilation was indicative of the onset and progression of DR. Benitez-Aguirre also found that VLF and vascular tortuosity can predict the occurrence of DR (33). To the best of our knowledge, this study is the first to analyze the changes in retinal arterial and venous vascular metrics in T1DM patients using OCTA images with a deep learning model. By employing vessel segmentation via a deep learning model, we provide a valuable supplement to the built-in metrics of the OCTA equipment to better explore the effect of DR on large vessels from a new perspective.

The intraretinal arteries are notably deficient in autonomic nerve supply. The integrity of the retinal arterial wall relies on multilayered smooth muscle cells that surround the artery and regulate the diameter of the lumen by contracting or relaxing in response to metabolic demands and blood flow (34). To compensate for the reduction in blood flow due to capillary nonperfusion in the early stages of diabetes, the smooth muscle cells of the arteries offer compensation through vasodilation (35), which manifests as an increase in VD. Our study found that in the group of patients with T1DM early in the disease duration (less than 5 years), the arterial VD of the inferior parafovea showed a compensatory increase. This finding has potential clinical applications in the management and monitoring of T1DM patients. Though being a retrospective study, the observed increase in arterial VD could serve as an early biomarker for vascular changes in T1DM, allowing for earlier intervention and prevention of further complications. Metrics representing the complexity of vessels, such as VLF and FD, exhibited an increasing trend, while VDI decreased. We hypothesized that the vascular structural changes induced by hyperglycemia were not uniform. The distribution of vascular smooth muscle cells, course of blood vessels, and hemodynamics may lead to uneven alterations of the vascular lumen (36). Some studies have also reported that the prevalence of diabetic retinopathy is higher in women than in men. Recently, it has also been found that at the NPDR stage, the area of the avascular zone in the macula and the superficial vascular curvature were more severe in women (37). This suggests that it is important for future research to investigate the vascular differences between genders. In addition, the obtained VDI specifically refers to the outer diameter of the blood vessel (6). This parameter ignores the thickness of the vascular wall and cannot accurately reflect the early changes in the inner diameter of the vessels.

However, with the gradual increase in disease duration, we did not observe a continuous alteration of arterial VD in the NDR group. We postulate that capillary expansion results in an increase in capillary VD as pericyte loss in the capillaries intensifies (38). Consequently, the compensation of the arteries disappears at this stage.

With the emergence of NPDR, veins in the inferior parafoveal zone have exhibited a reduction in vascular density (VD), whereas the arterial VD of the superior parafovea has begun to decline. However, we did not observe any significant changes in the VD of the inferior parafoveal arteries. Considering the denser distribution of the superior and inferior parafoveal vessels, the metabolism of retinal cells in this area is vigorous and more dependent on blood glucose (39). When blood glucose levels fluctuate, vascular blood flow alterations in this region are more sensitive. Vascular metric changes in veins have long been considered to be related to DR progression (19, 40). Our findings showed that a reduction in venous VD of the inferior parafovea had already occurred in the early stages of DR. These findings might potentially serve as a biomarker for identifying patients at risk of DR progression before visible retinal changes occur, enabling earlier intervention and management. However, longitudinal studies are required to investigate this possibility.

Previously, several researches have pointed out that SCP vessel tortuosity has shown significant changes during the NDR and NPDR stage, might be used as an early predictor for the occurrence and progression of DR (23, 41). In the present study, only a similar trend of changes in the parameters related to vascular curvature was found, without significant differences. This may be related to the methods used in this study. The vascular segmentation model applied in this study can only segment large and medium-sized vessels, and small vessels such as capillaries are not included in the calculation of vascular tortuosity-related parameters. Since diabetic retinopathy is characterized by microvascular changes in the first place, these small vessels contribute more to vessel tortuosity, and thus these parameters did not show statistically significant differences in the results of our study.

However, following limitations should be taken into consideration: (1) this study is a retrospective study and only includes a small number of samples; (2) There is an imbalance in the sample sizes among different groups; (3) clinical characteristics such as HbAc1 were not included, lacking relevant analysis; (4) OCTA imaging only scanned 3*3 mm region, unable to observe peripheral retinal vessels; and (5) vessel segmentation can only divide large vessels, not small vessels.

In conclusion, we employed a deep learning vessel segmentation model to analyze the changes in arterial and venous metrics in the OCTA images of patients with T1DM for the first time. We discovered that vascular damage occurs in the inferior parafoveal vein and superior parafoveal artery during the NPDR stage, which manifests as a reduction in vascular density. In patients with early T1DM without DR, there is a compensatory increase in the VD of the inferior parafoveal artery, potentially serving as an imaging biomarker for the early detection of DR.

## Data Availability

The datasets presented in this article are not readily available because the use of dataset requires the consent of the original author. Requests to access the datasets should be directed to NT, 13073288159@163.com.
