# Supplementary material for: Retinal vessel metric analysis of type 1 diabetes mellitus in OCT angiography
Source: Front Med (Lausanne). 2025 Jun 13;12:1562809. doi: 10.3389/fmed.2025.1562809 (PMC12202362; doi:10.3389/fmed.2025.1562809)
Supplement: Supplementary file 1 [file Table_1.docx]

| **Supplemental Table 1. Analysis of OCTA Equipment Built-in Metrics** | | | | |
| --- | --- | --- | --- | --- |
|  | Control (n = 70) | NDR (n = 91) | NPDR (n = 19) | p |
| superficial |  |  |  |  |
| fovea^a^ | 19.150 (14.275,22.325) | 18.200 (13.500,22.700) | 12.600 (11.000,16.100) | 0.005** |
| para-t^a^ | 50.450 (48.775,52.175) | 50.000 (47.800,51.500) | 46.400 (44.200,48.600) | <0.001*** |
| para-s | 53.724 ± 2.721 | 52.748 ± 2.737 | 48.711 ± 3.263 | <0.001*** |
| para-n | 51.294 ± 2.299 | 50.878 ± 2.515 | 46.932 ± 3.493 | <0.001*** |
| para-i^a^ | 53.050 (51.375,55.300) | 53.300 (50.800,54.700) | 50.300 (46.300,51.800) | <0.001*** |
| deep |  |  |  |  |
| fovea^a^ | 34.300 (25.600,37.600) | 33.300 (27.000,38.200) | 24.600 (21.000,26.300) | <0.001*** |
| para-t | 56.570 ± 2.363 | 56.012 ± 2.966 | 51.605 ± 3.243 | <0.001*** |
| para-s^a^ | 57.500 (55.400,58.950) | 56.600 (54.500,57.900) | 52.700 (51.400,54.300) | <0.001*** |
| para-n | 56.725 ± 1.950 | 56.100 (54.100,58.400) | 53.200 (50.900,54.700) | <0.001*** |
| para-i | 56.136 ± 2.641 | 55.479 ± 2.945 | 52.226 ± 3.990 | <0.001*** |
| FAZ^a^ | 0.308 (0.2290.394) | 0.299 (0.239,0.378) | 0.383 (0.333,0.459) | 0.017* |
| FD | 52.505 ± 3.012 | 52.130 ± 2.832 | 47.665 ± 4.787 | <0.001*** |
| NDR = non-diabetic retinopathy, NPDR = non-poliferative diabetic retinopathy, FAZ = foveal avascular zone, FD = fractal dimension  *p < 0.05, **p < 0.01, ***p < 0.001  ^a^The data were not normally distributed and variance was not even, p-values were obtained by nonparametric tests. | | | | |
