# Supplementary material for: Retinal vessel metric analysis of type 1 diabetes mellitus in OCT angiography
Source: Front Med (Lausanne). 2025 Jun 13;12:1562809. doi: 10.3389/fmed.2025.1562809 (PMC12202362; doi:10.3389/fmed.2025.1562809)
Supplement: Supplementary file 2 [file Table_2.docx]

| **Supplemental Table 2. Analysis of Vessel Metrics after Vessel Segmentation** | | | | |
| --- | --- | --- | --- | --- |
|  | Control (n = 70) | NDR (n = 91) | NPDR (n = 19) | p |
| arterial |  | | | |
| MDAC | 8.567 ± 1.32 | 8.772 ± 1.270 | 8.609 ± 0.981 | 0.514 |
| LC^a^ | 0.087 (0.071,0.098) | 0.088 (0.080,0.103) | 0.082 (0.075,0.097) | 0.225 |
| TSC-LX | 1.188 ± 0.190 | 1.210 ± 0.177 | 1.205 ± 0.184 | 0.716 |
| TSC-LC | 1.072 ± 0.169 | 1.086 ± 0.158 | 1.085 ± 0.161 | 0.837 |
| foveal^a^ | 0.001 (0.000,0.003) | 0.001 (0.000,0.002) | 0.000 (0.000,0.001) | 0.053 |
| para-t^a^ | 0.022 (0.016,0.029) | 0.023 (0.019,0.029) | 0.024 (0.019,0.027) | 0.451 |
| para-s | 0.033 ± 0.008 | 0.034 ± 0.010 | 0.029 ± 0.010 | 0.030* |
| para-n^a^ | 0.022 (0.018,0.026) | 0.022 (0.019,0.030) | 0.019 (0.013,0.026) | 0.154 |
| para-i | 0.032 ± 0.009 | 0.035 ± 0.009 | 0.034 ± 0.007 | 0.076 |
| FD | 0.859 ± 0.013 | 0.860 ± 0.013 | 0.856 ± 0.013 | 0.423 |
| VDI | 2.791 ± 0.163 | 2.794 ± 0.174 | 2.805 ± 0.153 | 0.933 |
| VLF | 0.010 ± 0.001 | 0.011 ± 0.001 | 0.010 ± 0.001 | 0.102 |
| venous |  |  |  |  |
| MDAC | 7.303 ± 1.175 | 7.245 ± 8.005 | 7.418 ± 0.931 | 0.760 |
| LC^a^ | 0.079 (0.067,0.096) | 0.076 (0.067,0.091) | 0.077 (0.068,0.092) | 0.926 |
| TSC-LX^a^ | 1.129 (1.038,1.238) | 1.159 (1.026,1.265) | 1.222 (1.026,1.274) | 0.680 |
| TSC-LC^a^ | 1.034 (0.954,1.120) | 1.051 (0.926,1.142) | 1.119 (0.916,1.170) | 0.733 |
| foveal^a^ | 0.001 (0.000,0.003) | 0.002 (0.000,0.003) | 0.001 (0.000,0.004) | 0.814 |
| para-t^a^ | 0.028 (0.024,0.035) | 0.028 (0.023,0.035) | 0.027 (0.023,0.032) | 0.719 |
| para-s^a^ | 0.040 (0.030,0.046) | 0.039 (0.033,0.050) | 0.036 (0.031,0.042) | 0.267 |
| para-n | 0.029 ± 0.009 | 0.030 ± 0.009 | 0.029 ± 0.010 | 0.528 |
| para-i^a^ | 0.040 (0.033,0.048) | 0.041 (0.031,0.050) | 0.035 (0.027,0.044) | 0.022* |
| FD | 0.863 ± 0.014 | 0.865 ± 0.013 | 0.864 ± 0.013 | 0.565 |
| VDI | 2.833 ± 0.256 | 2.862 ± 0.344 | 2.766 ± 0.244 | 0.361 |
| VLF^a^ | 0.013 (0.012,0.014) | 0.013 (0.012,0.015) | 0.013 (0.012,0.015) | 0.144 |
| NDR = non-diabetic retinopathy, NPDR = non-poliferative diabetic retinopathy, MDAC= mean direction angle change, LC= length of the curve, TSC-LX= total squared curvature normalized by L_x_, TSC-LC= total squared curvature normalized by Lc, FD= fractal dimension, VDI= vessel diameter index, VLF= vascular length fraction  *p < 0.05, **p < 0.01  ^a^The data were not normally distributed and variance was not even, p-values were obtained by nonparametric tests. | | | | |
