# Supplementary material for: Retinal vessel metric analysis of type 1 diabetes mellitus in OCT angiography
Source: Front Med (Lausanne). 2025 Jun 13;12:1562809. doi: 10.3389/fmed.2025.1562809 (PMC12202362; doi:10.3389/fmed.2025.1562809)
Supplement: Supplementary file 3 [file Table_3.docx]

| **Supplemental Table 3. Analysis of OCTA Built-in Metrics** | | | | |
| --- | --- | --- | --- | --- |
|  | Control (*n* = 70) | < 5y (*n* = 49) | ≥ 5y (*n* = 42) | p |
| superficial |  |  |  |  |
| fovea^a^ | 19.150 (14.275,22.325) | 17.600 (12.500,23.300) | 18.750 (15.550,21.900) | 0.998 |
| para-t^a^ | 50.450 (48.775,52.175) | 49.800 (48.000,51.200) | 50.350 (47.700,52.025) | 0.385 |
| para-s | 53.724 ± 2.721 | 52.792 ± 2.732 | 52.698 ± 2.775 | 0.083 |
| para-n | 51.294 ± 2.299 | 50.620 ± 2.511 | 51.179 ± 2.515 | 0.308 |
| para-i^a^ | 53.050 (51.375,55.300) | 52.600 (50.650,53.750) | 54.450 (50.950,55.500) | 0.029* |
| deep |  |  |  |  |
| fovea^a^ | 34.300 (25.600,37.600) | 32.300 (26.600,39.650) | 33.300 (27.150,35.900) | 0.599 |
| para-t | 56.570 ± 2.363 | 56.255 ± 2.703 | 55.729 ± 3.257 | 0.291 |
| para-s | 56.988 ± 2.698 | 56.576 ± 2.449 | 56.038 ± 2.971 | 0.201 |
| para-n | 56.725 ± 1.950 | 56.906 ± 2.814 | 55.829 ± 3.091 | 0.104 |
| para-i | 56.136 ± 2.641 | 55.647 ± 3.009 | 55.283 ± 2.892 | 0.289 |
| FAZ^a^ | 0.308 (0.229,0.394) | 0.307 (0.204,0.405) | 0.294 (0.244,0.358) | 0.997 |
| FD^a^ | 52.885 (50.480,54.523) | 53.440 (50.190,54.795) | 51.405 (49.653,53.270) | 0.063 |
| FAZ = foveal avascular zones, FD = fractal dimension  *p < 0.05, **p < 0.01  ^a^The data were not normally distributed and variance was not even, p-values were obtained by nonparametric tests. | | | | |
