# Supplementary material for: Retinal vessel metric analysis of type 1 diabetes mellitus in OCT angiography
Source: Front Med (Lausanne). 2025 Jun 13;12:1562809. doi: 10.3389/fmed.2025.1562809 (PMC12202362; doi:10.3389/fmed.2025.1562809)
Supplement: Supplementary file 4 [file Table_4.docx]

| **Supplemental Table 4. Analysis of Vessel Metrics after Vessel Segmentation** | | | | |
| --- | --- | --- | --- | --- |
|  | Control (n = 70) | < 5y (n = 49) | ≥ 5y (n = 42) | p |
| arterial |  | | | |
| MDAC | 8.548 ± 1.353 | 8.566 ± 0.907 | 8.710 ± 1.255 | 0.774 |
| LC^a^ | 0.085 (0.071,0.098) | 0.088 (0.080,0.105) | 0.085 (0.078,0.099) | 0.314 |
| TSC-LX | 1.177 ± 0.187 | 1.198 ± 0.187 | 1.217 ± 0.162 | 0.519 |
| TSC-LC | 1.063 ± 0.166 | 1.079 ± 0.169 | 1.091 ± 0.138 | 0.663 |
| foveal^a^ | 0.001 (0.000,0.003) | 0.001 (0.000,0.003) | 0.001 (0.000,0.002) | 0.893 |
| para-t^a^ | 0.022 (0.016,0.027) | 0.022 (0.017,0.029) | 0.023 (0.018,0.028) | 0.596 |
| para-s | 0.033 ± 0.008 | 0.033 ± 0.010 | 0.036 ± 0.011 | 0.270 |
| para-n^a^ | 0.022 (0.018,0.026) | 0.022 (0.020,0.032) | 0.021 (0.016,0.026) | 0.481 |
| para-i | 0.033 ± 0.009 | 0.037 ± 0.010 | 0.034 ± 0.009 | 0.035* |
| FD | 0.859 ± 0.012 | 0.860 ± 0.013 | 0.859 ± 0.014 | 0.900 |
| VDI | 2.792 ± 0.161 | 2.785 ± 0.166 | 2.803 ± 0.198 | 0.877 |
| VLF^a^ | 0.010 (0.009,0.011) | 0.011 (0.009,0.012) | 0.010 (0.009,0.011) | 0.152 |
| venous |  |  |  |  |
| MDAC | 7.336 ± 1.195 | 7.340 ± 0.981 | 7.291 ± 1.274 | 0.975 |
| LC^a^ | 0.079 (0.068,0.098) | 0.076 (0.069,0.091) | 0.080 (0.062,0.092) | 0..871 |
| TSC-LX | 1.136 ± 0.164 | 1.137 ± 0.142 | 1.158 ± 0.214 | 0.795 |
| TSC-LC | 1.027 ± 0.143 | 1.028 ± 0.131 | 1.040 ± 0.177 | 0.901 |
| foveal^a^ | 0.002 (0.000,0.003) | 0.002 (0.000,0.005) | 0.002 (0.001,0.003) | 0.826 |
| para-t^a^ | 0.029 (0.024,0.034) | 0.028 (0.023,0.035) | 0.030 (0.023,0.036) | 0.831 |
| para-s^a^ | 0.040 (0.030,0.046) | 0.043 (0.036,0.050) | 0.037 (0.031,0.050) | 0.103 |
| para-n | 0.029 ± 0.009 | 0.031 ± 0.010 | 0.030 ± 0.008 | 0.488 |
| para-i^a^ | 0.041 (0.034,0.047) | 0.041 (0.034,0.054) | 0.040 (0.031,0.045) | 0.475 |
| FD | 0.862 ± 0.014 | 0.865 ± 0.012 | 0.867 ± 0.013 | 0.201 |
| VDI | 2.830 ± 0.261 | 2.942 ± 0.409 | 2.816 ± 0.264 | 0.095 |
| VLF^a^ | 0.013 (0.012,0.014) | 0.013 (0.013,0.015) | 0.014 (0.012,0.015) | 0.195 |
| NDR = non-diabetic retinopathy, NPDR = non-poliferative diabetic retinopathy, MDAC= mean direction angle change, LC= length of the curve, TSC-LX= total squared curvature normalized by L_x_, TSC-LC= total squared curvature normalized by Lc, FD= fractal dimension, VDI= vessel diameter index, VLF= vascular length fraction  *p < 0.05, **p < 0.01  ^a^The data were not normally distributed and variance was not even, p values were obtained by nonparametric tests. | | | | |
